# Impact of Time-Restricted Eating and High-Intensity Exercise on Nutrient Intake in Women with Overweight/Obesity: Secondary Analysis of a Randomized Controlled Trial

**DOI:** 10.3390/nu17020218

**Published:** 2025-01-08

**Authors:** Kamilla L. Haganes, Brooke L. Devlin, Rosalie K. Orr, Trine Moholdt

**Affiliations:** 1 Department of Circulation and Medical Imaging, Faculty of Medicine and Health Sciences, Norwegian University of Science and Technology, 7491 Trondheim, Norway; trine.moholdt@ntnu.no; 2 Women’s Clinic, St. Olav’s Hospital, 7006 Trondheim, Norway; 3 School of Human Movement and Nutrition Sciences, Faculty of Health and Behavioural Sciences, The University of Queensland, Brisbane, QLD 4072, Australia; b.devlin@uq.edu.au (B.L.D.); rosalie.orr@hotmail.com (R.K.O.)

**Keywords:** time-restricted eating, high-intensity interval training, nutrition, overweight, obesity, diet quality

## Abstract

Background/Objectives: Inadequate micronutrient intakes are common in individuals with overweight/obesityand can exacerbate cardiovascular and metabolic disease risk. Diet and exercise are primary strategies for managing overweight and may influence nutrient intakes. In this secondary analysis of dietary data collected in a randomized controlled trial (RCT, ClinicalTrials.gov (NCT04019860), 15 June 2019) of time-restricted eating (TRE), high-intensity interval training (HIIT), a combination (TREHIIT), or a control group (CON), we investigated intervention effects on energy and nutrient intakes in women with overweight/obesity. Methods: We randomized 131 women (body mass index (BMI) ≥ 27 kg/m^2^) to 7 weeks of TRE (≤10-h daily eating window with ad libitum energy intake), HIIT (3 sessions/week, performed at ≥90% maximal heart rate), TREHIIT, or CON. Participants recorded all energy intake in an online food diary during a baseline week (week 0) and at the end of the study (week 6 and week 7). We investigated between-group differences in changes in mean energy, macronutrient, and micronutrient intakes. Results: TRE had reduced intakes of potassium, magnesium, and phosphorus compared with CON (*p* < 0.01). TREHIIT had non-significant reduced intakes of potassium, thiamine, magnesium, copper, and phosphorus (0.01< *p* < 0.05). HIIT alone did not negatively impact micronutrient intakes. TRE and TREHIIT induced suboptimal intakes for a greater number of micronutrients compared with HIIT and CON. Conclusions: A ≤10-h TRE window might increase the risk of micronutrient inadequacy in women with overweight/obesity. Future research is needed to investigate the effects of integrating nutritional guidelines with TRE interventions to mitigate the risk of micronutrient inadequacy in individuals with overweight/obesity.

## 1. Introduction

One in eight people are now living with obesity worldwide [1]. One of the primary drivers behind the global rise in obesity is an imbalance between energy intake and expenditure, often due to excessive energy consumption coupled with sedentary behavior [2]. Excessive energy intake commonly involves overconsumption of energy-dense foods with poor nutritive value and a simultaneous reduction in nutritious, minimally processed whole foods such as fruits, vegetables, whole grains, legumes, unprocessed meats, and dairy [3]. Hence, although obesity is often associated with “overnutrition”, people with overweight or obesity have more often inadequate micronutrient intakes compared with individuals with normal body weight [4,5].

Lifestyle interventions that focus on restrictingenergy intake and increasing energy expenditure through physical activity are primary approaches for weight loss and for improving cardiometabolic health. While energy-restricted diets are effective for weight loss, they carry the risk of worsening nutritional deficiencies [6]. As such, low-carbohydrate diets, intermittent fasting, and very low-calorie diets are associated with large and rapid weight loss, but may also increase the risk of inadequate nutrient intakes [6]. Adequate micronutrient intake is essential for physiological and metabolic health, as well as cognitive function, emotional well-being, and overall energy levels. Inadequate micronutrient intake in individuals with obesity can further increase the risk of cardiovascular and metabolic diseases [4,5]. For instance, inadequate intakes of vitamin D and magnesium, which play key roles in glucose metabolism and insulin signaling, are associated with an increased risk of developing type 2 diabetes [6,7,8,9].

Time-restricted eating (TRE) has gained popularity as a dietary strategy for weight loss and improved cardiovascular and metabolic health [10]. TRE aims to align energy intake with the body’s natural circadian rhythms, by restricting all energy intake to a limited daily eating window, without any deliberate alterations to total energy intake or dietary composition [11]. Despite no formal restrictions on total energy or dietary composition, TRE often induces spontaneous reductions in daily energy intake [12]. However, there is limited research on how ad libitum TRE without additional nutritional advice affects micronutrient intake [13]. Given the increased risk of nutrient inadequacy in individuals with overweight or obesity and its associated adverse health effects, studies are needed to understand how an altered meal timing influences dietary quality and micronutrient intake [6,11].

Physical exercise is the second cornerstone in the treatment of excess body fat stores, by increasing energy expenditure and promoting metabolic adaptations to support weight loss and cardiometabolic health. High-intensity interval training (HIIT) is an effective exercise strategy for improving cardiorespiratory fitness and reducing fat mass [14]. Intensive exercise can also acutely suppress appetite hormones, and exercise training may positively impact appetite regulation and reduce susceptibility to excessive energy intake [15,16]. Although some studies have shown shifts towards healthier dietary patterns after an HIIT intervention in young, normal-weight adults [17], others showed increased preference for high-energy, sugary foods, and reduced fiber intake after HIIT in middle-aged sedentary adults [18]. More research is needed on how high-intensity exercise influences total energy intake and nutrient composition. In this secondary analysis of a randomized controlled trial (RCT), we report the effects of 7 weeks of TRE, HIIT, or a combination (TREHIIT) versus a control group (CON) on energy, macronutrient, and micronutrient intakes in women with a body mass index (BMI) ≥ 27 kg/m^2^ [19].

## 2. Materials and Methods

### 2.1. Design and Participants

The protocol and primary results of the TREHIIT RCT have been published previously [19,20]. In brief, we recruited 131 women who were 18–45 years old and had a BMI ≥ 27 kg/m^2^. The participants were randomly allocated (1:1:1:1) to 7 weeks of TRE (*n* = 33), HIIT (*n* = 33), TRE and HIIT (TREHIIT, *n* = 32), or no intervention (CON, *n* = 33). The study duration was 8 weeks, with 1 week of baseline recordings and a 7-week intervention. All participants completed 1 week of baseline measurements (week 0) immediately after baseline laboratory assessments and randomization, before commencing the 7-week intervention period or 7 weeks of no intervention in CON.

We used a random number generator to allocate participants, as previously described [19]. Participants and study investigators were not blinded for group allocation. All the participants provided written informed consent, and the study was approved by the Regional Committee for Medical and Health Research Ethics in Middle Norway (REK no. 2019/851). The study was conducted in accordance with the Declaration of Helsinki and was registered at ClinicalTrials.gov (NCT04019860) on 15 June 2019.

### 2.2. Interventions

We instructed the participants allocated to TRE to limit their daily energy intake to a self-selected ≤10-h eating window, finishing no later than 20:00 h. Participants were allowed to consume non-energy-containing beverages during the fasting period (e.g., black coffee, water). We did not give any further dietary advice (e.g., total energy intake or food composition). Participants in the HIIT group performed three supervised exercise sessions per week of which two sessions were 4 × 4 min HIIT with work-bouts performed at 90–95% of maximal heart rate, and 1 session was 10 × 1 min HIIT, with work-bouts performed at the maximum intensity the participants could sustain for 1 min. The exercise sessions were performed on a treadmill or stationary bicycle in our laboratories. During the COVID-19 lockdown from March to August 2020, the exercise sessions were completed outside of the laboratory as running or uphill walking. The participants allocated to the TREHIIT group followed both protocols for TRE and HIIT. We instructed the participants in CON to maintain their habitual diet and physical activity levels throughout the entire 8-week study period. After completing post-assessments, we offered the participants in CON to choose one of the study interventions as a delayed treatment and receive 7 new weeks of follow-up. We did not collect any data from the delayed treatment period.

### 2.3. Dietary Intake Outcomes

We instructed the participants to register all energy intake in an electronic food diary (https://www.kostholdsplanleggeren.no/, accessed on 12 August 2019) every day during the first and last 14 days (week 0, week 1, week 6, and week 7) of the study. The online electronic food diary is a free online tool developed by the Norwegian Directorate of Health and The Norwegian Food Safety Authority. Users can search for and register food items from the integrated database. The database allows amounts to be registered as grams, dL, or portions. As such, participants could measure the amount of food using their preferred method. In cases when direct measurement of food was not possible, the participants were allowed to register estimated amounts. For food items not found in the database, participants could either manually register the food item and corresponding energy and nutritional content or choose an equivalent food item from the database. If we suspected that a participant had provided incomplete food registrations, for example only recording 500 kcal one day, the participant was contacted and asked if the registration was correct. The participants were allowed to send a new and updated diet diary if they had previously sent an incomplete diet diary.

In the analysis, we included baseline data from records during week 0 as an estimate of the participants’ habitual dietary intake and the average of recorded intake in weeks 6 and 7 as their intake during the intervention. The participants did not record their intake of dietary supplements (e.g., multivitamins, iron supplements, etc.).

We obtained the following dietary parameters from the food diaries: total energy intake (kJ and kcal), carbohydrates (g and energy percentage, E%), fiber (g), starch (g), added sugar (g and E%), fat (g and E%), saturated fat (g and E%), total cholesterol (mg), omega-3 fatty acids (g and E%), omega-6 fatty acids (g and E%), protein (g and E%), alcohol (g and E%), vitamin A (retinol activity equivalents), vitamin D (µg), vitamin E (alfa-TE), thiamine (mg), riboflavin (mg), niacin (mg), vitamin B6 (mg), folate (mg), vitamin B12 (µg), vitamin C (mg), calcium (mg), iron (mg), sodium (mg), potassium (mg), magnesium (mg), zinc (mg), selenium (µg), copper (mg), phosphorus (mg), and iodine (µg). Each dietary parameter was calculated as the average daily intake in week 0 and week 6 + 7. To calculate E%, we multiplied grams of fat, saturated fat, omega-3 fatty acids, and omega-6 fatty acids by 9 kcal/g, divided by total kcal intake, and multiplied by 100%. For carbohydrate, added sugar, and protein E%s, we multiplied grams by 4 kcal/g, divided by total kcal intake, and multiplied by 100%, while alcohol E% was determined by multiplying by 7 kcal/g.

### 2.4. Nutrient Intake Adequacy in Relation to the 2023 Nordic Nutrition Recommendations

We compared group means and individual participant’s mean daily nutrient intakes to reference values for healthy females aged 25–50 years provided in the 2023 Nordic Nutrition Recommendations (NNR2023) [21]. We used the estimated average requirement (EAR) to assess the risk of inadequate intake of micronutrients in the groups, as recommended by the NNR2023. The EAR is the estimated average daily nutrient intake needed to meet the requirements of half of the individuals in a specific life-stage group. We used the EAR as the cutoff for the following nutrients: vitamin A, vitamin D, riboflavin, niacin, vitamin B6, folate, vitamin C, calcium, iron, zinc, and copper. For nutrients for which the EAR cannot be determined, the NNR2023 provide a provisional average requirement (PAR), which is calculated by multiplying adequate intake (AI) by 0.8 [21]. We used the PAR as the cutoff for vitamin E, vitamin B12, phosphorus, potassium, magnesium, iodine, and selenium. For simplicity, we refer to both EAR and PAR as EAR from hereon. We defined a mean daily intake below the EAR as inadequate. We also present reference values for the recommended intake (RI) or AI. The RI is the average daily nutrient intake sufficient to meet the requirements of 97–98% of individuals in a particular life-stage group in the general population. AI is the observed or experimentally determined estimation of recommended daily nutrient intake assumed to be sufficient for a group of healthy individuals and is used when the RI for a nutrient cannot be established [21].

### 2.5. Statistics

We analyzed all participants in the TREHIIT trial following the intention-to-treat principle, regardless of the completeness of diet records. We used linear mixed models to investigate between-group differences in changes in mean nutrient intakes from baseline (week 0) to the end of the intervention period (weeks 6 and 7). In the model, we included time and the interaction between time and group (time × group) as fixed effects, while participant was included as a random effect. We adjusted for the baseline values, assuming no systematic effect on the group at baseline, as recommended by Twisk et al. [22], and inspected the normality of residuals by visually checking QQ plots. In cases of non-normality, we used bootstrapping with 3000 samples and bias-corrected and accelerated confidence intervals (CIs). Due to multiple comparisons, we considered two-sided *p*-values < 0.01 as statistically significant. Although some nutrients were non-normally distributed in one or more groups at baseline and/or at the end of the intervention, we present means and standard deviations (SDs) for average nutrient intakes, rather than median and percentile distributions, for clearer data presentation.

As inspired by Gardner et al. [23], we investigated the proportion of participants within each group with a shift in the risk of micronutrient inadequacy. We categorized participants who shifted from a micronutrient intake above the EAR at baseline (week 0) to below the EAR at the end of the intervention period (weeks 6 and 7) as “intake decreased below the EAR”. Participants who had micronutrient intakes above the EAR at both time points or below the EAR at both time points were categorized as “no change”, whereas those who shifted from a micronutrient intake below the EAR at baseline to above the EAR were categorized as “intake increased above the EAR”. We selected and display in a figure all micronutrients for which ≥20% of the participants in one or more groups fell into the category “intake decreased below the EAR”.

In the analysis of between-group differences in the proportions of participants falling within each category, we merged the two latter categories into “no change/improved micronutrient intake”. We used Fisher’s exact test to compare between-group differences in proportions since there were more than 20% of cells with expected counts below 5 for all micronutrients. For these comparisons, we present counts and percentages. For *p*-values < 0.01, we performed post hoc pairwise comparisons of proportions, with Bonferroni corrections. All statistical analyses were performed in IBM SPSS Statistics 29.0.1.0 and Stata/MP 18, while tables and figures were generated in Microsoft ^®^ Word Version 2404 and GraphPad Prism 9.

## 3. Results

### 3.1. Participants and Adherence to Interventions

We included 131 participants in the TREHIT trial, out of whom 21 were lost to follow-up. Table 1 shows the baseline characteristics of the participants, with more characteristics published previously [19]. Briefly, participants in TRE and TREHIIT reduced their eating window by ~3 h/day during the intervention period compared with baseline and had a ≤10-h eating window on ~6 days/week, as described in detail previously [19]. Participants in CON and HIIT did not alter their eating window and maintained a ~12-h eating window throughout the entire study period. Participants in HIIT and TREHIIT completed >90% of the 21 scheduled exercise sessions and completed the exercise sessions at >90% of individual maximal heart rate [19].

### 3.2. Total Energy and Macronutrients

There were no statistically significant between-group differences in total energy intake or E% outcomes after the 7-week intervention period (Table 2). TRE and TREHIIT reduced total energy intakes by 541 kJ (127 kcal) per day and 727 kJ (172 kcal) per day, respectively, but these changes were not statistically significantly different from CON (*p* = 0.051).

**Figure 1:**
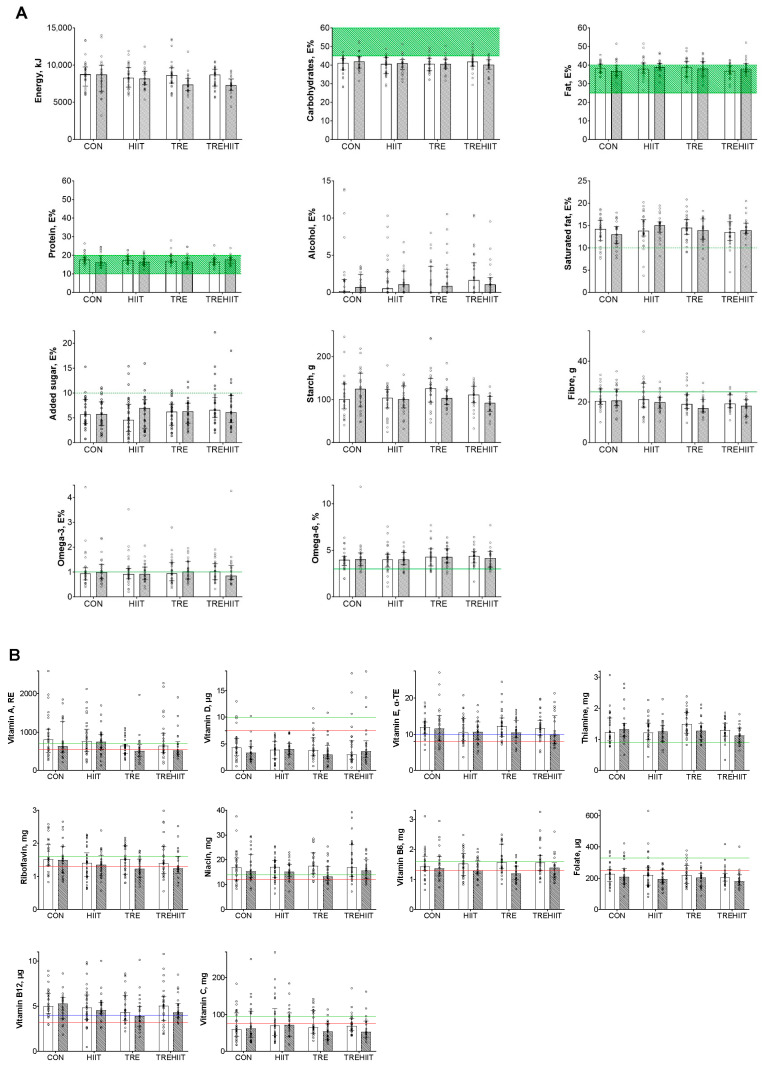

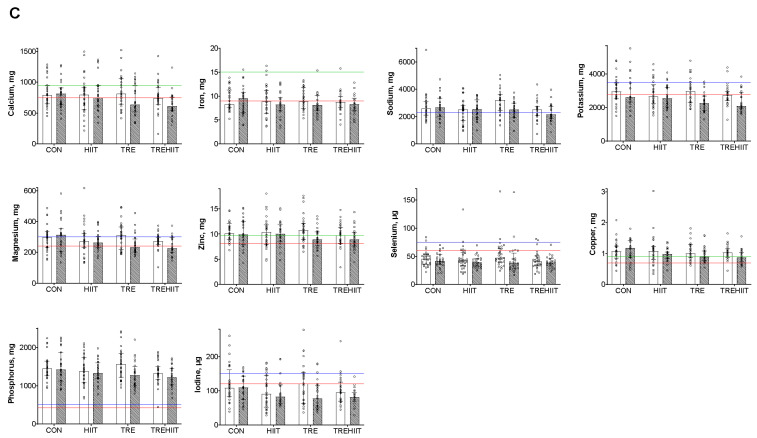
Median energy and nutrient intakes by group in relation to the 2023 Nordic Nutrition Recommendations. Vertical bars represent observed medians with interquartile ranges at baseline (blank) and at the end of the study (shaded) for the intention-to-treat population, according to group. Circles represent individual data points. Green shaded areas and green lines represent recommended intakes (RI) established by the 2023 Nordic Nutrition Recommendations (NNR2023). Red lines represent the estimated average requirements (EAR) or provisional average requirements (PAR), and blue lines represent adequate intakes (AI). The PAR was used as the cutoff for the following micronutrients: vitamin E, vitamin B12, phosphorus, potassium, magnesium, iodine, and selenium. (**A**) Descriptive statistics for total energy, macronutrients, and macronutrient energy percentages (E%s) outcomes, (**B**) descriptive statistics for vitamin outcomes, (**C**) descriptive statistics for mineral outcomes. Descriptive data at baseline are based on data from *n* = 28 in CON, *n* = 27 in TRE, *n* = 30 in HIIT, *n* = 27 in TREHIIT. Descriptive data at the end of the study are based on data from *n* = 23 in CON, *n* = 24 in TRE, *n* = 24 in HIIIT, *n* = 22 in TREHIIT. CON; control group, E%; energy percentage, HIIT; high-intensity interval training, kJ; kilojoules, RAE; retinol activity equivalents, α-TE; α-tocopherol equivalents, TRE; time-restricted eating, TREHIIT; time-restricted eating and high-intensity interval training. For clearer data visualization, one individual data point at y = 352.7 in the HIIT group at the end of the intervention was removed from the selenium graph.

There were indications of reduced carbohydrate intake in TRE (−21.6 g/day, *p* = 0.049) and in TREHIIT (−28.9 g/day, *p* = 0.010), and reduced fiber intake in TREHIIT (Table 2). There was a significant reduction in starch intake in TREHIIT (−26.0 g/day, *p* < 0.001). On average, the proportion of total energy coming from carbohydrates was below the RI of 45–60 E% in all groups at baseline and at the end of the intervention period, ranging between 40 and 42%. The fat E%s were within the RI of 25–40% in all groups (range 38–39%). The protein E%s were between 17% and 18%, also within the RI of 10–20 E% (Figure 1). Mean fiber intake was below the RI of ≥25–35 g/day in all groups (range 17–23 g/day) [21].

### 3.3. Micronutrient Intakes

In the TRE group, there were significantly reduced intakes of potassium, magnesium, and phosphorus at the end of the intervention period (all *p* < 0.01) and indications of reductions in thiamine and vitamin B6 (Table 2). The participants in the TREHIIT group reduced their intakes of potassium, thiamine, magnesium, copper, and phosphorus at the end of the intervention period, but these changes were not statistically significant compared with CON. The participants in HIIT increased their intake of vitamin D by 1.3 µg/day (*p* = 0.004), compared with CON. There were no significant between-group differences in any of the remaining nutrients (Table 2). Figure 1 shows that all groups had median micronutrient intakes below the EAR at baseline and at the end of the study for vitamin D, folate, vitamin C, and selenium. All groups had median iodine intakes below the EAR at the end of the study and at baseline, except for TRE, which had a median intake slightly above the EAR at baseline. At the end of the study, HIIT also had a median intake below the EAR for iron, while both TRE and TREHIIT had intakes below the EAR for vitamin A, riboflavin, calcium, iron, and magnesium. TRE also had an intake below the EAR for vitamin B6 at the end of the intervention.

The proportion of participants who changed from a micronutrient intake above the EAR at baseline to below the EAR at the end of the study (i.e., had an increased risk of micronutrient inadequacy) only differed between groups for vitamin A (*p* = 0.006). Post hoc comparisons revealed that the proportion of participants with a vitamin A intake that decreased below the EAR in TRE (43.5%) was different from CON (8.7%, *p* = 0.043) (Table 3).

**Figure 2:**
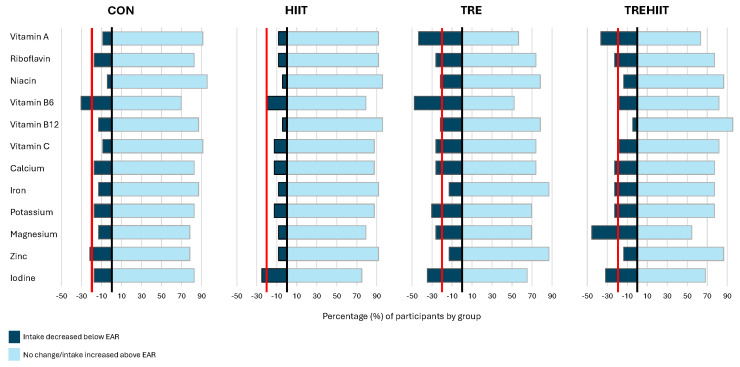
Proportions of participants with micronutrient intake decreasing below the EAR by group. The figure shows an overview of nutrients for which ≥20% of participants within one or more of the groups went from a mean intake above the EAR at baseline to below the EAR at the end of the study (intake decreased below the EAR). The red line shows the 20% cut-off. The horizontal bars show the percentage proportions of participants who shifted from a micronutrient intake above the EAR at baseline to below the EAR at the end of the study (dark blue) and the proportions who had no change/increased intake from below to above the EAR (light blue). Fisher’s exact test revealed a significant (*p* = 0.010) difference between groups for the proportions of participants with an intake decreasing below the EAR for Vitamin A. Post hoc group comparisons showed that the proportion with a Vitamin A intake decreasing below the EAR was greater in TRE (43.5%) compared with CON (8.7%, *p* = 0.043) and compared with HIIT (8.3%, *p* = 0.034). CON; control group, HIIT; high-intensity interval training, TRE; time-restricted eating, TREHIIT; time-restricted eating and high-intensity interval training.

Figure 2 shows micronutrients for which ≥20% of participants in one or more groups had a micronutrient intake shifting from above the EAR at baseline to below the EAR at the end of the intervention period. There were 10 micronutrients for which ≥20% of participants had intakes that decreased below the respective EARs at the end of the intervention in TRE, 7 micronutrients in TREHIIT, 2 in HIIT, and 2 in CON. The highest prevalence of an intake decreasing below the EAR was observed in TREHIIT for magnesium, in which 45.5% of the participants shifted from above the EAR to below the EAR.

## 4. Discussion

We investigated the effects of 7 weeks of TRE, HIIT, or a combination (TREHIIT) on nutrient intakes in women with a BMI ≥ 27 kg/m^2^. The interventions had no statistically significant effect on total energy intake or the distribution of macronutrients but affected the intake of some micronutrients. Participants allocated to TRE reduced their intake of potassium, magnesium, and phosphorus at the end of the intervention compared with participants in CON. There were additional indications of reduced intake of some other micronutrients in both the TRE and TREHIT groups, with a higher proportion of participants going from above to below the EARs.

All groups had mean intakes below the EAR throughout the study for vitamin D, folate, vitamin C, selenium, and iodine. Vitamin D deficiency is one of the most common micronutrient deficiencies among people with excess adiposity, with prevalence rates of >90% in adults with obesity, and is associated with an increased risk of type 2 diabetes [4,5]. The low folate intake across all groups is also of concern since the participants were women between 18 and 45 years. Folate deficiency in pregnant and reproductive-aged women increases the risk of infertility, cardiovascular disease, depression, and fetal neural tube defects [6]. Sources rich in folate are leafy green vegetables, legumes, and fortified grains [6]. A potential explanation for the low folate intake is the relatively high proportion of total energy coming from dietary fat, as higher-fat diets (>30 E%) are associated with decreased folate intake [24,25]. As such, all groups in our study had average fat E% intakes in the upper limit of the RI of 25–40 E%, ranging from 37 to 39 E% at baseline. Furthermore, all groups had saturated fat intakes above the RI, which is commonly observed in low-carbohydrate-high-fat diets [26].

Indeed, the amount of energy from carbohydrates was below the RI (45–60 E%) in all groups throughout the study. Our findings of lower-than-recommended carbohydrate intake, a high saturated fat intake, and up to 7.6 E% of added sugar on average, along with a low fiber intake, are in accordance with dietary patterns observed in the general Norwegian population. Almost 70% of women (69%) in the general Norwegian population reported carbohydrate intakes below the RI, a mean added sugar intake of 7.3 E%, and fiber intake below 25 g/day [27]. Low-carbohydrate-high-fat diets have received widespread popularity in the media as a weight-loss diet, which could potentially explain the macronutrient distributions observed in our study population, as well as in the general Norwegian population. Although low-carbohydrate diets induce weight loss and can improve glycemic control in individuals with insulin resistance or type 2 diabetes, there is a lack of evidence supporting that the overall benefits of a low-carbohydrate-high-fat diet outweigh the health effects of a diet higher in carbohydrates and lower saturated fat intake [26]. Diets low in carbohydrates are often deficient in fiber (which is found in fruits, vegetables, and whole grains), folate, potassium, calcium, magnesium, iron, vitamin A, and iodine [6,28]. The somewhat reduced intake of fiber, potassium, and magnesium in TREHIIT, as well as significant decreases in potassium and magnesium intakes in TRE, might thus partly be explained by reduced carbohydrate intakes in these groups. The lower carbohydrate intake in TRE might also underly the significantly larger proportion of participants with reduced vitamin A intake in TRE compared with CON.

Altering the daily eating window can influence the consumption of typical time-of-day-dependent foods despite allowing ad libitum intake during TRE [11,29,30]. In our study, participants in TRE and TREHIIT self-selected an eating window starting at 10:10 h and ending at 19:20 h on average [19], which may have reduced the intake of typical foods consumed for breakfast. Bread and cereals are common breakfast foods and are key carbohydrate sources in the Nordic countries [31]. While we did not analyze whether TRE affected specific food choices, it is possible that a delayed or skipped breakfast in the TRE and TREHIIT groups could partly explain the reduced intakes of carbohydrates and associated micronutrients.

Similar to our findings, an Australian study of individuals with type 2 diabetes who adhered to a 4-week ad libitum TRE intervention with the eating window from 10:00 h to 19:00 h reduced their intakes of carbohydrates, fiber, and potassium [30]. Another study from Turkey reported reduced intakes of several micronutrients (potassium, phosphorus, vitamin E, vitamin B2, vitamin B6, folate, vitamin C, and zinc) in women with BMI ≥ 25 kg/m^2^ after 8-h ad libitum TRE [32]. Other studies on late TRE have also reported reduced carbohydrate and added sugar intakes in American populations [33,34]. However, they did not report micronutrient intakes. Future studies are needed to understand how different timings of the eating window affect micronutrient intake, as carbohydrate sources typically consumed early in the day (e.g., fortified whole grains/cereals) might have different nutrition profiles than carbohydrate sources consumed in the late evening (e.g., pasta/rice). Notably, the influence of ad libitum TRE on macro- and micronutrient intakes might differ across cultures and countries due to differences in dietary patterns. For example, in Mediterranean countries, lunch accounts for a higher proportion (~38–45%) of the daily energy intake, compared with ~16–26% in countries in central and northern Europe. In central and northern European countries and in the U.S.A., dinner and after-dinner snacks account for the largest proportion of the daily energy intake [35,36].

Magnesium was also significantly reduced at the end of the intervention period for participants in the TRE group in our study. A substantial proportion, 26% of participants in TRE and 46% of those in TREHIIT changed their magnesium intake from above the EAR at baseline to below the EAR at the end of the intervention. Inadequate magnesium intake is prevalent among individuals with above-normal BMI, and reports show an inverse association between magnesium intake and excess adiposity [7]. Magnesium is involved in glucose oxidation pathways and influences the synthesis of triglycerides and low-density lipoprotein cholesterol. Additionally, magnesium plays an important role in vitamin D synthesis and helps optimize vitamin D status. As such, inadequate magnesium intake can exacerbate excess adiposity and increase the risk of dyslipidemia, metabolic syndrome, and type 2 diabetes [7]. Whole grains, nuts, seeds, legumes, and dark green vegetables are rich in magnesium, but supplementation might be necessary to meet the recommended intake of magnesium. Magnesium supplementation can improve blood pressure, lipid profile, and glycemic control in patients with type 2 diabetes and metabolic syndrome [7]. As such, maintaining an adequate intake of magnesium through dietary alterations or supplementation might be an important point of attention during energy restriction.

Engaging in exercise can affect energy intake and induce changes in food preferences through several physiological and behavioral mechanisms, which could subsequently affect nutrient intake. On the one hand, exercise can acutely suppress appetite hormones and improve appetite regulation, while on the other hand, some individuals may have an increased hedonic drive and reward value of energy-dense foods after completing exercise sessions [37,38]. While cumulative evidence does not show compensatory increases in energy intake after exercise, there are large inter-individual variations regarding exercise effects on eating behavior, which could result in overcompensation and lack of weight loss in some individuals after exercise interventions [38].

We reported unaltered appetite and unchanged total energy intake in the HIIT group, indicating no dietary compensation during the HIIT intervention in our study [19]. Our findings are in line with systematic reviews and meta-analyses reporting no change in energy intake after HIIT or other exercise interventions in individuals with overweight or obesity [15,39]. However, the participants in the HIIT group increased their vitamin D intake, suggesting a potential positive shift in dietary patterns. In contrast, a 12-week HIIT intervention induced negative changes in dietary quality in middle-aged sedentary adults, with increased intake of energy-dense foods and reduced fiber intake [18].

### 4.1. Clinical Implications

Ad libitum TRE can induce unintended reductions in total energy intake and concomitant weight loss. However, a poor baseline diet and poor dietary knowledge might limit the effectiveness of TRE and, in the worst case, exacerbate micronutrient inadequacy [40]. We observed that eating ad libitum within a ≤10-h time-restricted window can potentially worsen nutrient inadequacy, but it is not known whether the benefits of TRE outweigh the risk of nutrient inadequacy and associated health risks in adults with overweight or obesity. Notably, the risk of micronutrient inadequacy is not unique to TRE but is common with several energy-restrictive weight-loss diets [6,41]. While energy restriction is crucial for weight loss and for improving cardiovascular and metabolic health, it has been proposed that dietary interventions resulting in more gradual weight loss through portion control, the Mediterranean diet, or a low-glycemic-index diet, might be more appropriate to avoid worsening micronutrient deficiencies [41]. As such, there might be a potential synergic effect when integrating recommendations on what to eat with when to eat, but it is also important to consider how it affects long-term adherence. Allowing individuals to maintain their usual dietary preferences and not being required to monitor energy intake when undertaking TRE are some of the main facilitators of TRE adherence [40]. Future studies are needed to determine the feasibility of prescribing TRE in conjunction with existing dietary strategies and nutritional recommendations [11].

Phillips et al. [2021] conducted a study on 12-h ad libitum TRE versus a standard dietary advice group who received 10 min nutritional counseling at the beginning of the study in community-based adults with BMI ≥ 20 kg/m^2^ [42]. While they did not report micronutrient outcomes, they reported a reduced dietary proportion of ultra-processed foods and an increased proportion of unprocessed foods after 6 months in the standard dietary advice group versus the TRE group. In that study, they employed self-selected 12-h TRE, which might have been a too wide eating window to have a substantial effect on dietary composition and discretionary foods [42]. In another study on adults with type 2 diabetes, both an 8-h ad libitum TRE group (1200–2000 h) and a continuous caloric restriction group received regular dietary counseling, with advice on healthy food choices conforming to the American Diabetes Association nutrition guidelines. The study duration was 6 months, and the authors reported no difference over time or between groups in dietary intake and macronutrient composition, but they did not report micronutrient intakes. Nonetheless, adherence was higher in the TRE group (87% of days) compared with the continuous caloric restriction group (68% adhered to their prescribed kilocalorie goals), indicating that concomitant dietary advice might not have a major impact on long-term adherence to TRE [43]. Others have also provided nutritional counseling along with ad libitum TRE and reported relatively high adherence rates to the prescribed eating windows along with reductions in carbohydrate and added sugar intakes over 12 weeks [33,34]. The studies are limited by small sample sizes and did not report micronutrient intakes. As such, larger and more rigorous studies are needed to discern whether dietary counseling during a TRE intervention has an impact on dietary quality, long-term adherence, and health outcomes.

### 4.2. Strengths and Limitations

A strength of our study is that our participants recorded their intakes for 7 days at baseline and 14 days at the end of the study period, providing a large amount of data for estimating mean intakes, whereas many studies only collect data for a limited period (e.g., two weekdays and one weekend day). However, it can also be a limitation in that an increased participant burden can result in incomplete registrations. There are also other limitations related to the data collection method in our study. Participants recorded their daily dietary intake in an online food diary. The accuracy of the dietary data relies on the validity of the dietary database in the online food diary, and self-reporting is prone to under-reporting or incomplete registrations. Furthermore, inconsistencies in methods for estimating the amount and portions of food can affect the accuracy of the data. Some participants might have weighed and measured dietary intakes, while others may have visually estimated portion sizes. Additionally, reactivity is reported as an issue with dietary record keeping, i.e., changing usual dietary patterns to ease daily recording or to report foods perceived as healthy [44]. Another limitation is that some participants completed the study during the COVID-19 pandemic and lockdown periods, which also could have affected their dietary patterns. Participants could choose the timing of their first energy intake, provided their last energy intake was no later than 20:00 h and the eating window was ≤10 h/day. As such, variations in eating window timing within and between participants may have influenced the intake of time-of-day-dependent foods.

Other limitations are the lack of a separate sample size calculation to detect statistical differences between the groups for secondary outcomes and the risk of type 1 error due to multiple hypothesis testing. Furthermore, we used the EARs provided by the NNR2023 as cutoffs to evaluate nutrient adequacy in the groups. Mean group intakes below the EAR indicate less than 50% probability of adequate nutrient intake. However, intakes above the EAR do not indicate a high probability of adequate intake [21]. Therefore, there is a risk of underestimating nutrient inadequacy using these cutoff values. Regarding the evaluation of micronutrient inadequacy, a limitation of our study is that we did not obtain data on dietary supplement intake. Lastly, due to sociodemographic and cultural influences on dietary habits, our results might not be generalizable to other study populations or countries.

## 5. Conclusions

In this secondary analysis of dietary data from an RCT comparing 7 weeks of TRE, HIIT, a combination (TREHIIT), or a control group (CON), we found that ≤10-h TRE reduced intakes of certain micronutrients (potassium, magnesium, and phosphorus) compared with CON. HIIT alone did not have any significant negative effect on energy or micronutrient intakes. At the end of the study, there was an overall greater proportion of participants with an increased risk of micronutrient inadequacy in the TRE and TREHIIT groups compared with HIIT and CON. Our findings suggest that altering the timing of the daily eating window can have an impact on food composition and that integrating existing nutritional guidelines with TRE interventions might be important to mitigate the risk of micronutrient inadequacy in individuals with overweight or obesity.

## Figures and Tables

**Table 1:** Baseline characteristics of all randomized participants according to group. Data are for *n* participants in each group presented as descriptive means with standard deviations (SDs).

	CON (*n* = 33)		TRE (*n* = 33)		HIIT (*n* = 33)		TREHIIT (*n* = 32)	
	*n*	Mean (SD)	*n*	Mean (SD)	*n*	Mean (SD)	*n*	Mean (SD)
Age, years	33	36.4 (6.2)	33	36.2 (5.9)	33	34.9 (7.0)	32	37.3 (5.7)
Weight, kg	33	95.0 (11.2)	33	91.0 (10.8)	33	91.3 (13.0)	32	88.2 (10.3)
BMI, kg/m^2^	33	33.1 (4.2)	33	31.8 (3.3)	33	32.5 (4.5)	32	31.4 (4.0)
Total energy intake, kJ/day	28	8776.5 (1938.3)	27	8683.9 (2002.0)	30	8444.8 (1571.2)	27	8222.5 (1430.6)
Carbohydrates, E%	28	39.8 (4.9)	27	40.4 (20.4)	30	39.6 (5.0)	27	41.6 (4.7)
Fat, E%	28	38.0 (3.8)	27	38.0 (5.4)	30	38.6 (5.1)	27	37.0 (3.6)
Protein, E%	28	17.7 (3.1)	27	17.9 (3.0)	30	17.4 (2.6)	27	16.7 (2.6)
Alcohol, E%	28	2.2 (4.1)	27	1.6 (2.4)	30	1.9 (2.8)	27	2.5 (3.1)

BMI; body mass index, CON; control, E%; energy percentage, HIIT; high-intensity interval training, TRE; time-restricted eating, TREHIIT; time-restricted eating and high-intensity interval training.

**Table 2:** Intention-to-treat analyses of dietary parameters and micronutrient intakes. Observed data at baseline and at the end of the intervention for each group presented as descriptive means with standard deviations (SDs). The difference (group × time) is the mean change in the intervention group compared with the control group (CON), with estimate, corresponding 95% confidence intervals (95% CI), and *p*-values by linear mixed model analyses.

	Baseline (Week 0)	End of Intervention (Week 6 + 7)	Difference (Group × Time) Compared with CON		
	Mean (SD)	Mean (SD)	Estimated Effect	95% CI	*p*-Value
Energy and macronutrients					
Total energy intake, kJ/day					
CON	8776.5 (1938.3)	8503.9 (2523.8)			
TRE	8683.9 (2002.0)	7456.3 (1451.1)	−540.7	−1260.0 to 178.4	0.139
HIIT	8444.8 (1571.2)	8280.9 (1521.5)	153.5	−561.9 to 868.9	0.672
TREHIIT	8222.5 (1430.6)	7262.6 (1181.5)	−727.3	−1458.4 to 3.9	0.051
Total energy intake, kcal/day					
CON	2093.6 (465.5)	2028.6 (602.5)			
TRE	2072.5 (478.8)	1779.6 (346.7)	−127.3	−299.0 to 44.5	0.145
HIIT	2016.1 (374.5)	1977.7 (363.2)	39.4	−131.5 to 210.3	0.649
TREHIIT	1962.1 (341.2)	1733.3 (281.5)	−172.1	−346.7 to 2.6	0.053
Carbohydrates, g/day					
CON	210.9 (61.0)	213.1 (70.0)			
TRE	210.1 (53.5)	179.5 (37.5)	−21.6	−43.0 to −0.1	0.049
HIIT	200.6 (50.6)	200.7 (46.4)	−1.2	−22.5 to 20.2	0.913
TREHIIT	204.1 (42.6)	172.5 (37.3)	−28.9	−50.7 to −7.0	0.010
Carbohydrates, E%					
CON	39.8 (4.9)	41.8 (5.8)			
TRE	40.4 (20.4)	41.0 (4.4)	−1.5	−4.1 to 1.0	0.228
HIIT	39.6 (5.0)	40.8 (4.3)	−1.3	−3.9 to 1.2	0.291
TREHIIT	41.6 (4.7)	40.1 (4.5)	−2.5	−5.1 to 0.1	0.056
Fibre, g/day					
CON	22.1 (5.3)	22.3 (6.1)			
TRE	20.6 (5.8)	18.1 (4.4)	−2.0	−4.3 to 0.3	0.085
HIIT	22.6 (8.8)	20.0 (4.4)	−0.9	−3.1 to 1.4	0.459
TREHIIT	19.7 (3.9)	17.4 (4.3)	−2.6	−5.0 to −0.3	0.027
Starch, g/day					
CON	109.2 (46.3)	120.9 (48.0)			
TRE	126.3 (46.4)	107.2 (27.0)	−13.2	−28.1 to 1.8	0.083
HIIT	99.2 (35.0)	100.2 (32.1)	−7.5	−22.3 to 7.4	0.322
TREHIIT	107.0 (28.2)	89.4 (22.6)	−26.0	−41.2 to −10.8	<0.001
Added sugar, g/day					
CON	33.2 (20.4)	30.6 (20.1)			
TRE	30.7 (15.7)	28.4 (16.0)	−1.9	−10.2 to 6.4	0.652
HIIT	28.0 (23.0)	31.9 (18.3)	4.2	−4.0 to 12.5	0.312
TREHIIT	38.8 (27.4)	31.3 (21.1)	−1.8	−10.2 to 6.7	0.677
Added sugar, E%					
CON	6.1 (3.2)	5.9 (3.1)			
TRE	5.9 (2.6)	6.2 (2.7)	−0.0	−1.5 to 1.5	0.994
HIIT	5.3 (3.4)	6.4 (3.4)	0.9	−0.6 to 2.3	0.247
TREHIIT	7.6 (4.3)	7.0 (4.0)	0.3	−1.2 to 1.8	0.671
Fat, g/day					
CON	88.1 (19.3)	85.0 (28.9)			
TRE	88.1 (17.4)	76.2 (18.8)	−4.5	−12.9 to 3.9	0.292
HIIT	86.1 (17.4)	85.2 (15.6)	1.8	−6.7 to 10.1	0.673
TREHIIT	81.3 (18.1)	75.0 (13.2)	−4.9	−13.4 to 3.7	0.260
Fat, E%					
CON	38.0 (3.8)	37.3 (5.4)			
TRE	38.0 (5.4)	38.3 (4.7)	0.8	−1.5 to 3.0	0.496
HIIT	38.6 (5.1)	39.0 (3.6)	0.9	−1.4 to 3.1	0.438
TREHIIT	37.0 (3.6)	39.0 (4.0)	1.8	−0.5 to 4.0	0.117
Saturated fat, g/day					
CON	32.0 (11.7)	29.9 (12.0)			
TRE	33.5 (10.4)	27.5 (9.0)	−2.2	−6.1 to 1.7	0.261
HIIT	31.4 (10.1)	32.4 (7.5)	3.0	−0.9 to 6.8	0.133
TREHIIT	30.0 (9.4)	27.3 (7.3)	−0.5	−4.5 to 3.4	0.788
Saturated fat, E%					
CON	13.6 (3.2)	13.1 (2.8)			
TRE	14.5 (2.9)	13.7 (2.6)	0.0	−1.4 to 1.4	0.992
HIIT	14.0 (3.7)	14.8 (2.5)	1.3	−0.1 to 2.7	0.068
TREHIIT	13.6 (2.9)	14.2 (2.9)	1.2	−0.2 to 2.6	0.103
Cholesterol, mg/day					
CON	292.0 (102.5)	241.2 (90.5)			
TRE	292.0 (110.6)	241.3 (98.6)	3.1	−48.4 to 54.7	0.905
HIIT	276.7 (147.8)	260.1 (85.0)	37.6	−13.8 to 89.0	0.150
TREHIIT	278.8 (126.0)	282.7 (126.4)	44.2	−8.3 to 96.7	0.098
Omega-3, g/day *					
CON	2.4 (1.4)	2.4 (1.2)			
TRE	2.5 (1.2)	2.2 (0.9)	−0.1	−0.6 to 0.3	0.583
HIIT	2.3 (1.4)	2.1 (0.9)	−0.1	−0.6 to 0.4	0.736
TREHIIT	2.2 (0.9)	2.0 (1.4)	−0.2	−0.9 to 0.4	0.483
Omega-3, E% *					
CON	1.1 (0.8)	1.1 (0.5)			
TRE	1.1 (0.5)	1.1 (0.4)	0.0	−0.2 to 0.2	0.844
HIIT	1.0 (0.6)	1.0 (0.4)	0.0	−0.3 to 0.2	0.749
TREHIIT	1.0 (0.4)	1.1 (0.8)	0.0	−0.3 to 0.4	0.878
Omega-6, g/day *					
CON	9.1 (2.5)	9.8 (4.6)			
TRE	10.1 (3.9)	8.8 (2.7)	−1.3	−3.0 to 0.5	0.162
HIIT	8.9 (3.3)	8.8 (2.3)	−0.7	−2.5 to 1.1	0.456
TREHIIT	9.4 (2.8)	7.9 (2.2)	−2.1	−3.9 to −0.2	0.029
Omega-6, E% *					
CON	4.0 (1.0)	4.4 (1.8)			
TRE	4.4 (1.3)	4.4 (1.0)	−0.1	−0.9 to 0.6	0.752
HIIT	4.0 (1.4)	4.0 (0.9)	−0.3	−1.1 to 0.5	0.448
TREHIIT	4.3 (1.0)	4.2 (1.2)	−0.3	−1.2 to 0.5	0.413
Protein, g/day					
CON	90.4 (15.8)	85.5 (22.7)			
TRE	91.4 (19.4)	75.4 (16.4)	−7.3	−15.1 to 0.5	0.068
HIIT	87.1 (18.4)	82.7 (16.1)	0.6	−7.2 to 8.4	0.875
TREHIIT	81.5 (16.7)	75.7 (15.5)	−4.7	−12.6 to 3.3	0.248
Protein, E%					
CON	17.7 (3.1)	17.4 (3.6)			
TRE	17.9 (3.0)	17.1 (3.1)	−0.5	−1.7 to 0.7	0.371
HIIT	17.4 (2.6)	16.9 (2.3)	−0.4	−1.6 to 0.8	0.529
TREHIIT	16.7 (2.6)	17.5 (2.4)	0.4	−0.8 to 1.6	0.522
Alcohol, g/day *					
CON	6.8 (13.8)	3.6 (4.5)			
TRE	4.7 (6.9)	5.5 (8.5)	2.7	−1.2 to 6.5	0.172
HIIT	5.7 (8.8)	5.2 (6.8)	2.4	−1.1 to 5.8	0.183
TREHIIT	6.6 (8.4)	4.3 (5.1)	0.9	−2.2 to 3.9	0.573
Alcohol, E% *					
CON	2.2 (4.1)	1.2 (1.3)			
TRE	1.6 (2.4)	2.0 (2.9)	1.1	−0.1 to 2.4	0.069
HIIT	1.9 (2.8)	1.7 (2.0)	0.9	−0.1 to 1.9	0.075
TREHIIT	2.5 (3.1)	1.8 (2.3)	0.6	−0.5 to 1.7	0.309
Fat soluble vitamins					
Vitamin A, RAE/day *					
CON	904.4 (564.0)	789.2 (469.4)			
TRE	629.2 (220.1)	572.9 (349.2)	−104.8	−295.2 to 85.6	0.281
HIIT	845.0 (484.2)	761.6 (362.5)	40.0	−166.4 to 246.3	0.704
TREHIIT	814.1 (541.5)	662.3 (389.0)	−95.1	−316.5 to 126.3	0.400
Vitamin D, µg/day* ᵃ					
CON	4.8 (2.9)	3.8 (2.0)			
TRE	4.6 (2.5)	3.7 (2.4)	0.3	−0.7 to 1.2	0.583
HIIT	3.8 (2.0)	4.2 (1.6)	1.3	0.4 to 2.2	0.004
TREHIIT	4.7 (4.1)	5.1 (4.4)	1.3	−0.1 to 2.5	0.038
Vitamin E, α-TE/day					
CON	12.0 (3.0)	12.5 (5.7)			
TRE	12.5 (4.3)	10.9 (3.2)	−1.4	−3.2 to 0.4	0.127
HIIT	11.5 (3.9)	10.8 (3.4)	−1.1	−2.9 to 0.7	0.222
TREHIIT	12.1 (3.7)	11.3 (4.5)	−1.3	−3.2 to 0.5	0.158
Water soluble vitamins					
Thiamine, mg/day					
CON	1.4 (0.5)	1.4 (0.5)			
TRE	1.6 (0.4)	1.3 (0.4)	−0.2	−0.4 to 0.1	0.043
HIIT	1.3 (0.4)	1.2 (0.4)	−0.1	−0.2 to 0.1	0.375
TREHIIT	1.3 (0.3)	1.2 (0.3)	−0.2	−0.4 to 0.0	0.025
Riboflavin, mg/day					
CON	1.6 (0.5)	1.5 (0.5)			
TRE	1.5 (0.4)	1.2 (0.3)	−0.1	−0.3 to 0.1	0.193
HIIT	1.4 (0.5)	1.3 (0.4)	0.1	−0.1 to 0.3	0.416
TREHIIT	1.5 (0.6)	1.4 (0.4)	−0.1	−0.3 to 0.1	0.525
Niacin, mg/day					
CON	17.5 (6.7)	17.3 (6.3)			
TRE	18.6 (5.5)	14.8 (4.4)	−2.2	−4.8 to 0.5	0.109
HIIT	16.5 (4.7)	15.7 (3.8)	−0.5	−3.1 to 2.2	0.724
TREHIIT	19.4 (8.2)	16.2 (4.4)	−1.7	−4.4 to 1.0	0.207
Vitamin B6, mg/day					
CON	1.5 (0.5)	1.5 (0.5)			
TRE	1.7 (0.5)	1.2 (0.3)	−0.3	−0.5 to −0.1	0.015
HIIT	1.5 (0.4)	1.4 (0.3)	−0.1	−0.3 to 0.1	0.551
TREHIIT	1.7 (0.6)	1.4 (0.4)	−0.1	−0.4 to 0.1	0.190
Folate, µg/day					
CON	228.0 (65.5)	230.9 (81.5)			
TRE	226.8 (68.0)	197.7 (51.7)	−19.5	−47.2 to 8.2	0.165
HIIT	232.7 (109.7)	206.3 (54.4)	2.9	−24.6 to 30.4	0.832
TREHIIT	214.4 (57.9)	193.6 (58.7)	−20.1	−48.2 to 18.1	0.160
Vitamin B12, µg/day					
CON	5.4 (1.5)	5.0 (1.6)			
TRE	4.9 (1.9)	4.2 (1.9)	−0.3	−1.2 to 0.6	0.520
HIIT	5.0 (2.1)	4.9 (1.5)	0.2	−0.7 to 1.0	0.736
TREHIIT	5.0 (2.2)	4.8 (1.4)	−0.1	−1.0 to 0.8	0.893
Vitamin C, mg/day *					
CON	70.8 (39.8)	78.1 (55.2)			
TRE	77.4 (33.5)	55.9 (24.5)	−22.3	−43.0 to −1.7	0.034
HIIT	86.5 (59.4)	78.4 (42.9)	1.1	−22.9 to 25.2	0.926
TREHIIT	74.3 (27.7)	62.9 (33.7)	−15.3	−36.3 to 5.7	0.154
Minerals					
Calcium, mg/day					
CON	819.3 (235.3)	801.8 (231.5)			
TRE	849.9 (267.6)	701.4 (248.8)	−75.1	−185.8 to 35.6	0.182
HIIT	781.6 (302.0)	810.1 (277.6)	39.4	−70.8 to 149.6	0.481
TREHIIT	762.0 (241.6)	640.9 (201.9)	−112.8	−225.4 to −0.1	0.050
Iron, mg/day					
CON	9.2 (2.3)	9.1 (3.1)			
TRE	9.4 (2.2)	8.4 (2.3)	−0.4	−1.5 to 0.6	0.442
HIIT	9.1 (3.2)	8.3 (2.5)	−0.0	−1.1 to 1.0	0.979
TREHIIT	8.8 (2.2)	8.2 (2.1)	−0.5	−1.6 to 0.5	0.331
Sodium, mg/day					
CON	2711.0 (1005.0)	2691.5 (865.9)			
TRE	3099.3 (922.0)	2457.7 (703.7)	−320.2	−675.1 to 34.6	0.076
HIIT	2384.4 (872.1)	2509.7 (712.1)	63.3	−289.8 to 416.4	0.723
TREHIIT	2468.4 (720.0)	2271.6 (679.5)	−181.9	−542.8 to 178.9	0.320
Potassium, mg/day					
CON	3007.7 (816.1)	2996.0 (1002.2)			
TRE	2995.0 (788.1)	2362.7 (585.7)	−391.7	−661.9 to −121.6	0.005
HIIT	2803.7 (794.5)	2634.7 (690.0)	15.6	−252.8 to 284.0	0.909
TREHIIT	2740.9 (621.1)	2381.4 (617.5)	−326.0	−600.3 to −51.7	0.020
Magnesium, mg/day					
CON	295.1 (80.8)	306.8 (108.6)			
TRE	306.6 (86.5)	250.4 (69.6)	−45.2	−75.0 to −15.5	0.003
HIIT	282.5 (101.8)	270.9 (60.9)	−0.2	−29.7 to 29.3	0.989
TREHIIT	272.5 (50.5)	242.4 (59.4)	−37.6	−67.8 to −7.4	0.015
Zinc, mg/day					
CON	10.5 (2.0)	10.4 (2.9)			
TRE	11.0 (2.9)	9.2 (2.0)	−1.0	−2.2 to 0.2	0.093
HIIT	10.5 (3.3)	10.2 (2.4)	0.1	−1.1 to 1.3	0.913
TREHIIT	9.8 (2.3)	9.1 (2.3)	−0.8	−2.1 to 0.4	0.180
Selenium, µg/day *					
CON	46.8 (14.1)	43.2 (13.3)			
TRE	53.6 (26.9)	45.3 (29.7)	1.0	−9.3 to 11.2	0.851
HIIT	45.7 (21.9)	53.4 (64.6)	10.6	−16.1 to 37.3	0.437
TREHIIT	43.0 (15.7)	39.7 (10.70)	−3.1	−9.4 to 3.3	0.343
Copper, mg/day					
CON	1.1 (0.3)	1.1 (0.4)			
TRE	1.1 (0.3)	1.0 (0.3)	−0.1	−0.2 to 0.0	0.161
HIIT	1.1 (0.5)	1.0 (0.2)	−0.1	−0.2 to 0.1	0.440
TREHIIT	1.0 (0.2)	0.9 (0.2)	−0.2	−0.3 to 0.0	0.026
Phosphorus, mg/day					
CON	1484.0 (329.1)	1510.2 (430.0)			
TRE	1571.9 (406.2)	1296.3 (344.8)	−197.7	−326.8 to −68.7	0.003
HIIT	1414.1 (386.8)	1363.2 (296.1)	−34.7	−162.9 to 93.6	0.593
TREHIIT	1351.1 (312.1)	1224.1 (279.0)	−169.6	−300.7 to −38.6	0.012
Iodine, µg/day					
CON	119.7 (55.2)	107.6 (38.4)			
TRE	121.9 (60.3)	87.2 (43.1)	−21.0	−43.6 to 1.7	0.069
HIIT	98.4 (48.5)	97.0 (41.1)	−0.7	−23.3 to 21.9	0.953
TREHIIT	102.0 (41.9)	81.5 (25.0)	−19.7	−42.8 to 3.4	0.094

Descriptive data at baseline are based on data from *n* = 28 in CON, *n* = 27 in TRE, *n* = 30 in HIIT, *n* = 27 in TREHIIT. Descriptive data at the end of the study are based on data from *n* = 23 in CON, *n* = 24 in TRE, *n* = 24 in HIIIT, *n* = 22 in TREHIIT. The number of completely missing diet data (0 days registered) at baseline (week 0) was *n* = 5 in CON, *n* = 6 in TRE, *n* = 2 in HIIT, and *n* = 6 in TREHIIT. The number of completely missing diet data (0 days registered) in week 6 was *n* = 11 in CON, *n =* 9 in TRE, *n* = 9 in HIIT, *n* = 11 in TREHIIT, and *n* = 11 in CON, *n* = 13 in TRE, *n* = 8 in HIIT, and *n* = 11 in TREHIIT in week 7. The number of missing days in the dietary records at baseline (week 0) was 5 (2.6%) in CON (one participant with 4 missing days, one with 1 missing day), 2 (1.1%) in HIIT, and 5 (2.6%) in TREHIIT. The number of missing days during weeks 6 and 7 were 12 (3.7%) in CON, 7 (2.3%) in HIIT, 13 (4.0%) in TRE, and 14 (4.3%) in TREHIIT. Between-group analyses are based on all randomized participants in the TREHIIT trial, regardless of missing data (*n* = 33 in CON, *n* = 33 in TRE, *n* = 33 in HIIT, *n* = 32 in TREHIIT). CON; control, E%; energy percentage, HIIT; high-intensity interval training, kcal; kilocalories, kJ; kilojoules, RAE; retinol activity equivalents, α-TE; α-tocopherol equivalents, TRE; time-restricted eating, TREHIIT; time-restricted eating and high-intensity interval training. * 95% CI and *p*-values are from bootstrap with 3000 samples and bias-corrected and accelerated confidence intervals due to non-normally distributed residuals.

**Table 3:** Between-group differences in the proportions of participants with a shift in the risk of micronutrient inadequacy at the end of the study. Total number and percentage proportions of participants with unchanged/increased and decreased mean nutrient intakes at the end of the study, in relation to the estimated average requirements (EARs) provided in the 2023 Nordic Nutrition Recommendations (NNR2023), according to group. We used the provisional average requirement (PAR) as the cutoff for vitamin E, vitamin B12, phosphorus, potassium, magnesium, iodine, and selenium. Participants who had no change in micronutrient intake or increased intake to above the EAR (going from below the EAR at baseline to above at the end of the intervention) were categorized together as “no change/intake increased above the EAR”, while participants who went from a micronutrient intake above the EAR at baseline to below the EAR at the end of the intervention period were categorized as “intake decreased below the EAR”. *p*-values are for Fisher’s exact tests. Post hoc pairwise comparisons were only conducted for Fisher’s exact test *p*-values < 0.01.

Vitamins	EAR	No Change/Intake Increased Above EAR, No. (%)	Intake Decreased Below EAR, No. (%)	*p*-Value Fisher’s Exact Test
Vitamin A, RAE/day	540.0			0.006
CON		21 (91.3%)	2 (8.7%)	
HIIT		22 (91.7%)	2 (8.3%)	
TRE		13 (56.5%)	10 (43.5%)	0.043 vs. CON 0.034 vs. HIIT
TREHIIT		14 (63.6%)	8 (36.4%)	
Vitamin D, µg/day	7.5			0.112
CON		20 (87.0%)	3 (13.0%)	
HIIT		24 (100%)	0	
TRE		21 (91.3%)	2 (8.7%)	
TREHIIT		22 (100%)	0	
Vitamin E, α-TE/day	8.0			0.695
CON		21 (91.3%)	2 (8.7%)	
HIIT		22 (91.7%)	2 (8.3%)	
TRE		20 (87.0%)	3 (13.0%)	
TREHIIT		18 (81.8%)	4 (18.2%)	
Thiamine, mg/day	0.65			0.740
CON		22 (95.7%)	1 (4.3%)	
HIIT		24 (100.0%)	0	
TRE		23 (100.0%)	0	
TREHIIT		22 (100.0%)	0	
Riboflavin, mg/day	1.3			0.393
CON		19 (82.6%)	4 (17.4%)	
HIIT		22 (91.7%)	2 (8.3%)	
TRE		17 (73.9%)	6 (26.1%)	
TREHIIT		17 (77.3%)	5 (22.7%)	
Niacin, mg/day	12.0			0.213
CON		22 (95.7%)	1 (4.3%)	
HIIT		23 (95.8%)	1 (4.2%)	
TRE		18 (78.3%)	5 (21.7%)	
TREHIIT		19 (86.4%)	3 (13.6%)	
Vitamin B6, mg/day	1.3			0.128
CON		16 (69.6%)	7 (30.4%)	
HIIT		19 (79.2%)	5 (20.8%)	
TRE		12 (52.2%)	11 (47.8%)	
TREHIIT		18 (81.8%)	4 (18.2%)	
Folate, µg/day	250.0			0.887
CON		21 (91.3%)	2 (8.7%)	
HIIT		22 (91.7%)	2 (8.3%)	
TRE		20 (87.0%)	3 (13.0%)	
TREHIIT		19 (86.4%)	3 (13.6%)	
Vitamin B12, µg/day	3.2			0.221
CON		20 (87.0%)	3 (13.0%)	
HIIT		23 (95.8%)	1 (4.2%)	
TRE		18 (78.3%)	5 (21.7%)	
TREHIIT		21 (95.5%)	1 (4.5%)	
Vitamin C, mg/day	75.0			0.420
CON		21 (91.3%)	2 (8.7%)	
HIIT		21 (87.5%)	3 (12.5%)	
TRE		17 (73.9%)	6 (26.1%)	
TREHIIT		18 (81.8%)	4 (18.2%)	
Minerals				
Calcium, mg/day	750.0			0.651
CON		19 (82.6%)	4 (17.4%)	
HIIT		21 (87.5%)	3 (12.5%)	
TRE		17 (73.9%)	6 (26.1%)	
TREHIIT		17 (77.3%)	5 (22.7%)	
Iron, mg/day	9.0			0.596
CON		20 (87.0%)	3 (13.0%)	
HIIT		22 (91.7%)	2 (8.3%)	
TRE		20 (87.0%)	3 (13.0%)	
TREHIIT		17 (77.3%)	5 (22.7%)	
Potassium, mg/day	2800.0			0.501
CON		19 (82.6%)	4 (17.4%)	
HIIT		21 (87.5%)	3 (12.5%)	
TRE		16 (69.6%)	7 (30.4%)	
TREHIIT		17 (77.3%)	5 (22.7%)	
Magnesium, mg/day	240.0			0.015
CON		20 (78.3%)	3 (13.0%)	
HIIT		22 (79.2%)	2 (8.3%)	
TRE		17 (69.6%)	6 (26.1%)	
TREHIIT		12 (54.5%)	10 (45.5%)	
Zinc, mg/day	8.1			0.638
CON		18 (78.3%)	5 (21.7%)	
HIIT		22 (91.7%)	2 (8.3%)	
TRE		20 (87.0%)	3 (13.0%)	
TREHIIT		19 (86.4%)	3 (13.6%)	
Selenium, µg/day	60.0			0.929
CON		22 (95.7%)	1 (4.3%)	
HIIT		21 (87.5%)	3 (12.5%)	
TRE		21 (91.3%)	2 (8.7%)	
TREHIIT		20 (90.9%)	2 (9.1%)	
Copper, mg/day	0.7			0.402
CON		20 (87.0%)	3 (13.0%)	
HIIT		23 (95.8%)	1 (4.2%)	
TRE		21 (91.3%)	2 (8.7%)	
TREHIIT		18 (81.8%)	4 (18.2%)	
Phosphorus, mg/day	420.0			-
CON		23 (100%)	0	
HIIT		24 (100%)	0	
TRE		23 (100%)	0	
TREHIIT		22 (100%)	0	
Iodine, µg/day	120.0			0.568
CON		19 (82.6%)	4 (17.4%)	
HIIT		18 (75.0%)	6 (25.0%)	
TRE		15 (65.2%)	8 (34.8%)	
TREHIIT		15 (68.2%)	7 (31.8%)	

The analyses are based on data from participants with complete dietary data: *n* = 23 in CON, *n* = 24 in TRE, *n* = 24 in HIIIT, *n* = 22 in TREHIIT. CON; control, the EAR; estimated average requirement, HIIT; high-intensity interval training, RAE; retinol activity equivalents, α-TE; α-tocopherol equivalents, TRE; time-restricted eating, TREHIIT; time-restricted eating and high-intensity interval training.

## Data Availability

Deidentified participant data underlying the results in this article are available from the corresponding author upon reasonable request.

## References

[1] World Health Organization. Obesity and Overweight. https://www.who.int/news-room/fact-sheets/detail/obesity-and-overweight.

[2] Swinburn B.A., Sacks G., Hall K.D., McPherson K., Finegood D.T., Moodie M.L., Gortmaker S.L. (2011). The global obesity pandemic: Shaped by global drivers and local environments. Lancet.

[3] Kant A.K. (2003). Reported consumption of low-nutrient-density foods by American children and adolescents: Nutritional and health correlates, NHANES III, 1988 to 1994. Arch. Pediatr. Adolesc. Med..

[4] Agarwal S., Reider C., Brooks J.R., Fulgoni V.L. (2015). Comparison of prevalence of inadequate nutrient intake based on body weight status of adults in the United States: An analysis of NHANES 2001–2008. J. Am. Coll. Nutr..

[5] Astrup A., Bügel S. (2019). Overfed but undernourished: Recognizing nutritional inadequacies/deficiencies in patients with overweight or obesity. Int. J. Obes..

[6] Bradley M., Melchor J., Carr R., Karjoo S. (2023). Obesity and malnutrition in children and adults: A clinical review. Obes. Pillars.

[7] Piuri G., Zocchi M., Della Porta M., Ficara V., Manoni M., Zuccotti G.V., Pinotti L., Maier J.A., Cazzola R. (2021). Magnesium in Obesity, Metabolic Syndrome, and Type 2 Diabetes. Nutrients.

[8] Wimalawansa S.J. (2018). Associations of vitamin D with insulin resistance, obesity, type 2 diabetes, and metabolic syndrome. J. Steroid Biochem. Mol. Biol..

[9] Zakharova I., Klimov L., Kuryaninova V., Nikitina I., Malyavskaya S., Dolbnya S., Kasyanova A., Atanesyan R., Stoyan M., Todieva A. (2019). Vitamin D Insufficiency in Overweight and Obese Children and Adolescents. Front. Endocrinol..

[10] Manoogian E.N.C., Chow L.S., Taub P.R., Laferrère B., Panda S. (2022). Time-restricted Eating for the Prevention and Management of Metabolic Diseases. Endocr. Rev..

[11] Parr E.B., Devlin B.L., Hawley J.A. (2022). Perspective: Time-Restricted Eating-Integrating the What with the When. Adv. Nutr..

[12] Ezpeleta M., Cienfuegos S., Lin S., Pavlou V., Gabel K., Tussing-Humphreys L., Varady K.A. (2024). Time-restricted eating: Watching the clock to treat obesity. Cell Metab..

[13] Varady K.A., Cienfuegos S., Ezpeleta M., Gabel K. (2022). Clinical application of intermittent fasting for weight loss: Progress and future directions. Nat. Rev. Endocrinol..

[14] Campbell W.W., Kraus W.E., Powell K.E., Haskell W.L., Janz K.F., Jakicic J.M., Troiano R.P., Sprow K., Torres A., Piercy K.L. (2019). High-Intensity Interval Training for Cardiometabolic Disease Prevention. Med. Sci. Sports Exerc..

[15] Beaulieu K., Blundell J.E., van Baak M.A., Battista F., Busetto L., Carraça E.V., Dicker D., Encantado J., Ermolao A., Farpour-Lambert N. (2021). Effect of exercise training interventions on energy intake and appetite control in adults with overweight or obesity: A systematic review and meta-analysis. Obes. Rev..

[16] Hu M., Kong Z., Shi Q., Nie J. (2023). Acute effect of high-intensity interval training versus moderate-intensity continuous training on appetite-regulating gut hormones in healthy adults: A systematic review and meta-analysis. Heliyon.

[17] Donati Zeppa S., Sisti D., Amatori S., Gervasi M., Agostini D., Piccoli G., Bertuccioli A., Rocchi M.B.L., Stocchi V., Sestili P. (2020). High-intensity Interval Training Promotes the Shift to a Health-Supporting Dietary Pattern in Young Adults. Nutrients.

[18] Jurado-Fasoli L., Amaro-Gahete F.J., De-la-O A., Castillo M.J. (2020). Impact of different exercise training modalities on energy and nutrient intake and food consumption in sedentary middle-aged adults: A randomised controlled trial. J. Human. Nutr. Diet..

[19] Haganes K.L., Silva C.P., Eyjólfsdóttir S.K., Steen S., Grindberg M., Lydersen S., Hawley J.A., Moholdt T. (2022). Time-restricted eating and exercise training improve HbA1c and body composition in women with overweight/obesity: A randomized controlled trial. Cell Metab..

[20] Moholdt T., Silva C., Lydersen S., Hawley J. (2021). Isolated and combined effects of high-intensity interval training and time-restricted eating on glycaemic control in reproductive-aged women with overweight or obesity: Study protocol for a four-armed randomised controlled trial. BMJ Open.

[21] Blomhoff R., Andersen R., Arnesen E.K., Christensen J.J., Eneroth H., Erkkola M., Gudanaviciene I., Halldorsson T.I., Høyer-Lund A., Lemming E.W. (2023). Nordic Nutrition Recommendations 2023.

[22] Twisk J., Bosman L., Hoekstra T., Rijnhart J., Welten M., Heymans M. (2018). Different ways to estimate treatment effects in randomised controlled trials. Contemp. Clin. Trials Commun..

[23] Gardner C.D., Kim S., Bersamin A., Dopler-Nelson M., Otten J., Oelrich B., Cherin R. (2010). Micronutrient Quality of Weight-loss Diets That Focus on Macronutrients: Results From the A TO Z Study. Am. J. Clin. Nutr..

[24] Hampl J.S., Betts N.M. (1995). Comparisons of dietary intake and sources of fat in low- and high-fat diets of 18- to 24-year-olds. J. Am. Diet. Assoc..

[25] Xanthakos S.A. (2009). Nutritional deficiencies in obesity and after bariatric surgery. Pediatr. Clin. North. Am..

[26] Brouns F. (2018). Overweight and diabetes prevention: Is a low-carbohydrate-high-fat diet recommendable?. Eur. J. Nutr..

[27] Lundblad M.W., Andersen L.F., Jacobsen B.K., Carlsen M.H., Hjartåker A., Grimsgaard S., Hopstock L.A. (2019). Energy and nutrient intakes in relation to National Nutrition Recommendations in a Norwegian population-based sample: The Tromsø Study 2015–16. Food Nutr. Res..

[28] Crowe T.C. (2005). Safety of low-carbohydrate diets. Obes. Rev..

[29] Gill S., Panda S. (2015). A Smartphone App Reveals Erratic Diurnal Eating Patterns in Humans that Can Be Modulated for Health Benefits. Cell Metab..

[30] Parr E.B., Devlin B.L., Lim K.H.C., Moresi L.N.Z., Geils C., Brennan L., Hawley J.A. (2020). Time-Restricted Eating as a Nutrition Strategy for Individuals with Type 2 Diabetes: A Feasibility Study. Nutrients.

[31] Engeset D., Hofoss D., Nilsson L.M., Olsen A., Tjønneland A., Skeie G. (2015). Dietary patterns and whole grain cereals in the Scandinavian countries--differences and similarities. The HELGA project. Public. Health Nutr..

[32] Mengi Çelik Ö., Köksal E., Aktürk M. (2023). Time-restricted eating (16/8) and energy-restricted diet: Effects on diet quality, body composition and biochemical parameters in healthy overweight females. BMC Nutr..

[33] Bakhsh J.A., Vu M.H., Salvy S.J., Goran M.I., Vidmar A.P. (2024). Effects of 8-h time-restricted eating on energy intake, dietary composition and quality in adolescents with obesity. Pediatr. Obes..

[34] Hegedus E., Vu M.H., Salvy S.J., Bakhsh J., Goran M.I., Raymond J.K., Espinoza J.C., Vidmar A.P. (2024). Randomized Controlled Feasibility Trial of Late 8-h Time-Restricted Eating for Adolescents with Type 2 Diabetes. J. Acad. Nutr. Diet..

[35] Kant A.K., Graubard B.I. (2015). 40-Year Trends in Meal and Snack Eating Behaviors of American Adults. J. Acad. Nutr. Diet..

[36] Huseinovic E., Winkvist A., Slimani N., Park M.K., Freisling H., Boeing H., Buckland G., Schwingshackl L., Weiderpass E., Rostgaard-Hansen A.L. (2016). Meal patterns across ten European countries—Results from the European Prospective Investigation into Cancer and Nutrition (EPIC) calibration study. Public. Health Nutr..

[37] Finlayson G., Bryant E., Blundell J.E., King N.A. (2009). Acute compensatory eating following exercise is associated with implicit hedonic wanting for food. Physiol. Behav..

[38] King N.A., Horner K., Hills A.P., Byrne N.M., Wood R.E., Bryant E., Caudwell P., Finlayson G., Gibbons C., Hopkins M. (2012). Exercise, appetite and weight management: Understanding the compensatory responses in eating behaviour and how they contribute to variability in exercise-induced weight loss. Br. J. Sports Med..

[39] Taylor J., Keating S.E., Holland D.J., Coombes J.S., Leveritt M.D. (2018). The Chronic Effect of Interval Training on Energy Intake: A Systematic Review and Meta-Analysis. J. Obes..

[40] O’Connor S.G., Boyd P., Bailey C.P., Shams-White M.M., Agurs-Collins T., Hall K., Reedy J., Sauter E.R., Czajkowski S.M. (2021). Perspective: Time-Restricted Eating Compared with Caloric Restriction: Potential Facilitators and Barriers of Long-Term Weight Loss Maintenance. Adv. Nutr..

[41] Chapela S., Martinuzzi A., Llobera N.D., Ceriani F., González V., Montalván M., Verde L., Frias-Toral E. (2024). Obesity and micronutrients deficit, when and how to suplement. Food Agric. Immunol..

[42] Phillips N.E., Mareschal J., Schwab N., Manoogian E.N.C., Borloz S., Ostinelli G., Gauthier-Jaques A., Umwali S., Gonzalez Rodriguez E., Aeberli D. (2021). The Effects of Time-Restricted Eating versus Standard Dietary Advice on Weight, Metabolic Health and the Consumption of Processed Food: A Pragmatic Randomised Controlled Trial in Community-Based Adults. Nutrients.

[43] Pavlou V., Cienfuegos S., Lin S., Ezpeleta M., Ready K., Corapi S., Wu J., Lopez J., Gabel K., Tussing-Humphreys L. (2023). Effect of Time-Restricted Eating on Weight Loss in Adults With Type 2 Diabetes: A Randomized Clinical Trial. JAMA Netw. Open.

[44] Bailey R.L. (2021). Overview of dietary assessment methods for measuring intakes of foods, beverages, and dietary supplements in research studies. Curr. Opin. Biotechnol..

